# Brazilian Cochrane Center: 15^th^ Anniversary

**DOI:** 10.1590/S1516-31802012000600010

**Published:** 2013-01-18

**Authors:** Rachel Riera, Álvaro Nagib Atallah, Tamara Melnik, Maria Regina Torloni

**Affiliations:** I MD, PhD. Professor, Postgraduate Program on Internal Medicine and Therapeutics, Universidade Federal de São Paulo, São Paulo, Brazil. Researcher, Brazilian Cochrane Center, São Paulo, Brazil.; II MD, PhD. Full Professor, Division of Emergency and Evidence-Based Medicine; Coordinator, Postgraduate Program on Internal Medicine and Therapeutics, Universidade Federal de São Paulo, São Paulo, Brazil. Director, Brazilian Cochrane Center, São Paulo, Brazil.; III BSc, PhD. Psychologist and Professor, Postgraduate Program on Internal Medicine and Therapeutics, Universidade Federal de São Paulo, São Paulo, Brazil. Researcher, Brazilian Cochrane Center, São Paulo, Brazil.

Last year, the Brazilian Cochrane Center (BCC) celebrated its 15^th^ anniversary!

We would like to thank all BCC members and numerous external collaborators, as well as several Brazilian government institutions, for their generous and unrestricted support throughout these years.

We are very pleased to present a brief summary of the main accomplishments of the BCC since its inauguration in 1996, under the thoughtful gaze of Sir Ian Chalmers.

The Cochrane Library became freely available to users in Brazil, and later this was extended to all Latin America and Caribbean countries. This was possible thanks to an initial partnership with the Pan-American Health Organization, which has now been replaced by a partnership with CAPES (Coordenação de Aperfeiçoamento de Pessoal de Nível Superior; Brazilian Coordinating Agency for Improvement of University-level Personnel).

In cooperation with the postgraduate program on Internal Medicine and Therapeutics of Universidade Federal de São Paulo, over these years, the BCC has also served as a “research laboratory” and teaching center for more than 300 graduate students in health-related programs. The Center has helped students and university professors to produce Cochrane Systematic Reviews that have contributed to master’s and doctoral theses and professorship titles.

Since December 2002, the BCC website has offered a free online course (in Portuguese) that provides basic information and concepts on how to prepare systematic reviews and conduct meta-analyses. The number of hits on the main page of this course reached 12,276 between March and August 2010. The main BCC webpage receives more than 1,500,000 hits per year.

Over the years, the BCC team of research assistants has conducted dozens of high-quality systematic reviews commissioned by the Brazilian Ministry of Health, following the Cochrane methodology and standards. Cochrane estimates indicate that these reviews have saved the country’s public health system an average of 5 billion United States dollars per year.

Since 2007, the BCC has been offering a year-long Continuing Medical Education course on Evidence-Based Healthcare through a videoconferencing system (two hours/week). This free national course is available for remote regions of the country and was made possible thanks to a successful partnership between the BCC, the Brazilian Ministry of Health and the research department of a private hospital (Hospital Sírio-Libanês). The BCC team is responsible for organizing, coordinating and teaching this course. Over 10,000 healthcare professionals and policymakers have been trained through this course up to 2011 and many relevant research projects have been developed.

The BCC team offers continuing education and training to public servants within the Brazilian Health Technology Assessment Network (REBRATS), an agency that works for the Brazilian Ministry of Health’s Department of Science, Technology and Strategic Supplies.

The BCC broadcasts a weekly one-hour TV show, to more than 30 open channels in the whole country, presenting scientific evidence directed both towards healthcare and legal professionals and towards patients as well.

A few years ago, the BCC had the honor of organizing the first Cochrane Colloquium ever held in Latin America, an event that congregated over 800 participants from dozens of countries and contributed towards bringing the Cochrane Collaboration to the attention of Brazilian consumers and healthcare professionals. Recently, members of BCC were closely involved (Dr. Álvaro Atallah was the scientific chairman) in a national meeting of the Scientific Committee for Health Technology Assessment, held in Rio de Janeiro, in 2011. It was a unique opportunity to disseminate the methods of the Cochrane Collaboration to professionals in charge of Health Technology Assessment in several countries.

Since 2008, the BCC has established important partnerships with bodies within the field of Law (National Council of Justice, General Attorney’s Office and National Council of Public Prosecutors) through formal technical-institutional cooperation agreements. The objectives of this new cooperation are to improve lawyers’ and judges’ education and knowledge regarding health-related topics and to help them use the best available evidence in reaching verdicts and imposing sentences.

Through cooperation with Associação Paulista de Medicina (APM) and with the agreement of the Cochrane Collaboration, several abstracts of Cochrane Systematic Reviews have been published in São Paulo Medical Journal/Evidence for Health Care, followed by independent comments from Brazilian specialists, in the section “Cochrane Highlights”. A similar section has been introduced in Revista Diagnóstico & Tratamento, another journal that is supported by APM.

In recognition of the work done by the BCC, the President of Brazil, Luis Inácio Lula da Silva, visited our institution in 2010.

On March 29, 2011 through ordinance number 625, the Brazilian Ministry of Health published a proposal for “Guidelines for Adaptation of Clinical Practice Guidelines”, which highlights that Cochrane Systematic Reviews should be considered to be the main evidence in the process of producing guidelines in Brazil.

Throughout these years, the BCC has strived to disseminate high-quality knowledge and to construct strong bonds of trust and respect with several national partners, with the main objective of creating a new culture of Evidence-Based Medicine (now Evidence-Based Healthcare) in Brazil. The reward for these efforts culminated last year, with the promulgation of a federal law (number 12,401) on April 28, 2011. This law mandates that the National Council for Incorporation of Technologies should base its recommendations for incorporation of new technologies into the Brazilian national health system on scientific evidence of efficacy, effectiveness, safety and accuracy, as well as comparative economic assessment of benefits and costs. Although the importance and significance of this federal law are uncontestable, we know that our work is still at the beginning.

Finally, we would like to express our sincere gratitude to all those who stood by us throughout these 15 years and who contributed to the growth and consolidation of the Brazilian Cochrane Center and to its main objective, which is the definitive implementation of a culture of Evidence-Based Healthcare in Brazil, through the development and dissemination of Cochrane Systematic Reviews.

To view the text of the Brazilian Cochrane Center’s 15^th^ Anniversary statement published online at The Cochrane Collaboration website, please access the link: http://www.cochrane.org/features/brazilian-cochrane-centre-turns-15.

